# Prevalence and proportion of *Plasmodium* spp. triple mixed infections compared with double mixed infections: a systematic review and meta-analysis

**DOI:** 10.1186/s12936-020-03292-8

**Published:** 2020-06-24

**Authors:** Manas Kotepui, Kwuntida Uthaisar Kotepui, Giovanni D. Milanez, Frederick R. Masangkay

**Affiliations:** 1 Medical Technology, School of Allied Health Sciences, Walailak University, Tha Sala, Nakhon Si Thammarat, Thailand; 2 Department of Medical Technology, Far Eastern University-Manila, Manila, Philippines

**Keywords:** *Plasmodium*, Mixed infection, Triple infection, Quadruple infection, Concurrent infection

## Abstract

**Background:**

Although mixed infection by two *Plasmodium* species has been recognized, mixed infection by three different *Plasmodium* species within one individual has not been clarified. This study sought to determine the pooled prevalence and proportion of triple mixed *Plasmodium* spp. infection compared with double mixed infection.

**Methods:**

Articles from PubMed, Scopus, and Web of Science were searched for cross-sectional studies of triple mixed infection by *Plasmodium* species and then were retrieved and extracted. The pooled proportion and prevalence of triple mixed infection by *Plasmodium* species were subjected to random-effects analysis. The secondary outcomes were differences in the pooled proportion between triple mixed infection and double mixed infection by *Plasmodium* species reported in the included studies.

**Results:**

Of 5621 identified studies, triple mixed infection data were available for 35 records, including 601 patients from 22 countries. The overall pooled prevalence of triple mixed infection was 4% (95% Confidence Interval (CI) 3–5%; I^2^ = 92.5%). The pooled proportion of triple mixed infection compared with double mixed infection was 12% (95% CI 9–18; I^2^ = 91%). Most of the included studies (29/35; 82.9%) presented a lower proportion of triple mixed infection than double mixed infection. Subgroup analysis demonstrated that the proportion of triple mixed infection was the highest in Oceania (23%; 95% CI 15–36%) and Europe (21%; 95% CI 5–86%), but the lowest in the USA (3%; 95% CI 2–4%). Moreover, the proportion of triple mixed infection was higher in residents (20%; 95% CI 14–29%) than in febrile patients (7%; 95% CI 4–13%), when compared with the proportion of double mixed infection. Subgroup analysis of the age groups demonstrated that, compared with the proportion of double mixed infection, triple mixed infection was lower in patients aged ≤ 5 years (OR = 0.27; 95% CI 0.13–0.56; I^2^ = 31%) and > 5 years (OR = 0.09; 95% CI 0.04–0.25, I^2^ = 78%).

**Conclusions:**

The present study suggested that, in areas where triple mixed infection were endemic, PCR or molecular diagnosis for all residents in communities where malaria is submicroscopic can provide prevalence data and intervention measures, as well as prevent disease transmission and enhance malaria elimination efforts.

## Background

Malaria remains a major disease of public health concern worldwide, particularly in sub-Saharan Africa [1]. In 2018, the World Health Organization (WHO) estimated 228 million cases and 405,000 deaths caused by malaria worldwide, mostly in children aged younger than 5 years [1]. Infections of the *Plasmodium* species usually present as monoinfection by one species; however, mixed infections by more than one species within one individual can occur [2–6]. The interactions between mixed infections are not well characterized, but may play roles in disease progression and outcomes [7]. Moreover, mixed infections by *Plasmodium* species are often not recognized or are underestimated by microscopists [8]. In Asia, mixed infections by *Plasmodium* species have occurred at a frequency of 2% to 30% [9]. Although mixed infection by two *Plasmodium* species has been recognized, mixed infection by three different *Plasmodium* species within one individual has not been clarified.

This study sought to determine the pooled prevalence and proportion of triple mixed infection by *Plasmodium* spp. compared with double mixed infection. This information is necessary to guide the progress of research on mixed infection and malaria management, as well as control strategies for strategic malaria diagnostic service choices and treatment options.

## Methods

### Search strategy

Articles from PubMed, Scopus, and Web of Science were searched for cross-sectional studies on triple mixed infection by *Plasmodium* species in patients with all species of malaria. Triple mixed infection were defined as infection with three *Plasmodium* species. Articles published between February 2, 1907, and February 24, 2020, in the English language were included in the analysis if they explicitly reported the presence of triple mixed infection by *Plasmodium* species. The search strategy included the search terms “(Plasmodium OR Malaria) AND (“Mixed infection” OR “Triple infection”)” (Additional file 1: Table S1).

### Selection criteria

Observational studies, prospective cohorts, and case–control designs were included if they reported triple mixed malaria infection among the included participants by polymerase chain reaction (PCR) or molecular methods. Studies were excluded if the numbers of triple mixed infection could not be extracted and if only one species of *Plasmodium* was studied or evaluated subsequently from microscopy or rapid diagnostic test (RDT). Animal studies, clinical drug trials, case reports, experimental studies, reviews, systematic reviews, and polymorphism studies were excluded because they were considered incompatible study designs for the present review and meta-analysis. Studies were selected and identified by two independent authors (MK and KUK), with discrepancies resolved following discussion with a third author (FM). The protocol of this analysis was reported according to the Preferred Reporting Items for Systematic Reviews and Meta-Analyses (PRISMA) guidelines.

### Data extraction and definitions

The data extracted for individual studies included author names, year of publication, study area, year of study, details, and numbers of the participants, age ranges, blood collection methods, DNA extraction method, investigated gene, PCR method, malaria positivity status, number of double mixed infections, and number of triple mixed infections. The numbers of patients with four different *Plasmodium* species were also extracted for further discussions in the present study. Subgroup analyses were performed for the following parameters: publication year (before and after 2000), continent (Asia, Africa, America, Europe, or Oceania), participant group (febrile patients and residents from the same community), type of blood storage for PCR (EDTA blood or dried blood spots), and age group of patients with mixed infection.

### Data analysis

The primary outcome was the pooled proportion and pooled prevalence of triple mixed infection by *Plasmodium* species, with random-effects meta-regression used to investigate these pooled analyses. The analyses were performed using Stata Statistical Software (Release 15; StataCorp LLC. USA). The secondary outcomes were differences in the pooled proportion between triple mixed infection and dual mixed infection by *Plasmodium* species reported in the included studies. Those differences were estimated using random-effects meta-analysis to calculate the odds ratio (ORs) and 95% confidence intervals (CI). The analyses were performed using Review Manager 5 (RevMan 5, Cochrane Community).

Potential bias related to individual studies was assessed using a tool developed by the Newcastle-Ottawa Scale (NOS) to assessing the quality of non-randomized studies in meta-analyses. The quality of included studies was rated if they qualified with a maximum of 7 stars. Publication bias related to study effects was assessed by funnel plot asymmetry. Between-study heterogeneity was assessed by the I^2^ statistic and was assessed using the random-effects statistic. Subgroup analysis of the baseline characteristics included continent (Asia, Africa, America, Europe, or Oceania), participants (residents and febrile individuals), type of blood storage for PCR (EDTA blood or dried blood spots), and age group. For the subgroup analyses of age groups and different mixed infections (double and triple infection), the age groups of patients were classified as ≤ 5 and > 5 years because children younger than 5 years are one of the most vulnerable groups affected by malaria [10].

## Results

### Characteristics of the included studies

**Fig. 1:**
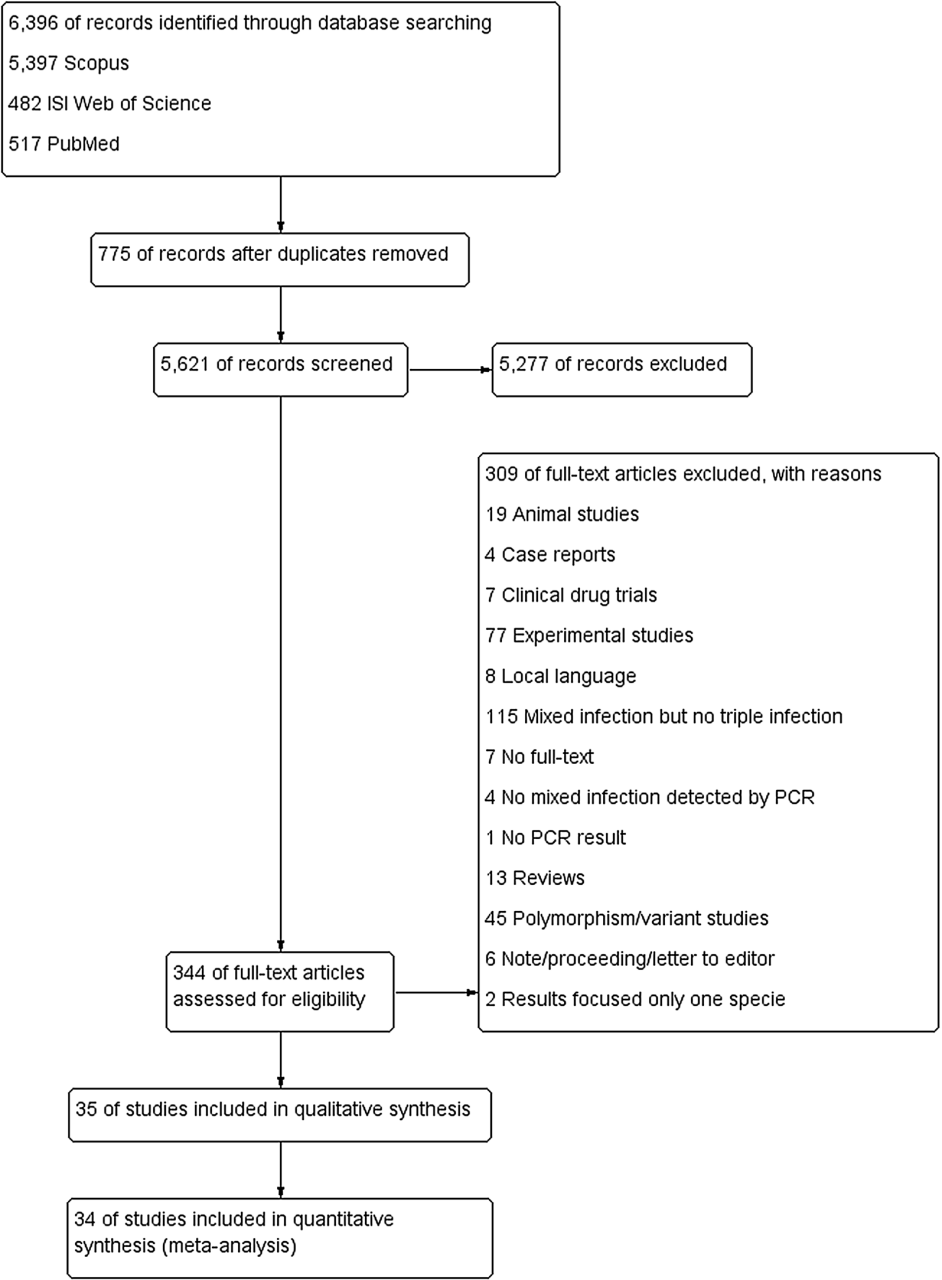
Flow chart for the study selection

After screening the titles and abstracts of 5621 identified studies published between February 2, 1907, and February 24, 2020, the full texts of 344 (6.11%) potentially relevant studies were reviewed (Fig. 1). There were 309 studies that did not meet the inclusion criteria, mostly because they did not document triple mixed infection by *Plasmodium* species in their studies. Thirty-five (10.1%) of 344 studies could be extracted and were included in the analysis (Table 1). Of the 35 studies, 14 (40%) were from the Asia–Pacific region, 9 (25.7%) were from Africa, 5 (14.3%) were from Europe, 4 (11.4%) were from Oceania, and three (8.6%) were from the (Table 1). Among 35 included studies from 22 different countries, most (4/35, 11.4%) were from Papua New Guinea [11–14], Cambodia [15–17], India [6, 18, 19], Italy [4, 20, 21], and Uganda [2, 22, 23]. Most of the participants included among the studies were residents (16/35, 45.7%), febrile patients (11/35, 31.4%), and malaria-positive cases (6/35, 17.1%). Twenty-four studies (68.6%) reported age ranges, whereas others did not. One study used samples from doubtful microscopic examination [18], whereas another enrolled both febrile and asymptomatic patients to perform PCR analysis [6]. More than half of the included studies (18/35, 51.4%) used EDTA blood to extract the DNA for PCR analysis, whereas others used dried blood spots (15/35, 42.9%), and one study used thick smears for DNA extraction [24]. Most of the studies (28/35, 80%) used DNA commercial kits, while four studies (4/35, 11.4%) used 30% Chelex-100 and phenol–chloroform extraction for DNA extraction. All of the included studies used the *18S ribosomal RNA* (rRNA) gene to identify the *Plasmodium* genus and species. Overall, 44,310 participants were enrolled in the included studies. Of those, most were residents (34,483, 77.8%), febrile patients (7797, 17.6%), and malaria-positive samples (1675, 3.78%).

**Table 1:** Characteristics of the included studies

No.	Author, year	Study area (years of the survey)	Participants	Age range	Age groups (n)	Blood storage for PCR	DNA extraction	Investigated gene	PCR method	Malaria positive by PCR	Mixed infection (dual infection)	Mixed infection (Triple infection)
1.	Asua et al. [2]	Uganda	Malaria positive (499)	6 months to 10 years		Dried blood spots	Chelex extraction kits	18S rRNA	Nested PCR	474	Pf/Pm (19) Pf/Po (14) Pf/Pv (3) Pm/Pv (1) Total = 37	Pf/Pm/Po (1)
2.	Barber et al. [3]	Malaysia (2009–2011)	Malaria positive (653)	*P. knowlesi* (IQR 20–50 years), *P. falciparum* (IQR 9–31 years), *P. vivax* (19 years, IQR 7–32 years)	Triple infection: < 5 (1), 10–14 (1), 30–34 (1), 80–84 (1) Dual infection: < 5 (1), 5–9 (2), 10–14 (5), 15–19 (3), 20–24 (2), 25–29 (2), 30–34 (4), 35–39 (3), 40–44 (5), 45–49 (2), 50–54 (2), 70–74 (2), 75–79 (1)	NA	NA	18S rRNA	Nested PCR	445	Pv/Pk (36) Pf/Pk (6) Pm/Pk (1) Pf/Pm (1) Total = 44	Pf/Pv/Pk (2) Pv/Pm/Pk (2)
3.	Calderaro et al. [4]	Italy (2000–2007)	Febrile patients (701)	NA		EDTA blood	High Pure Template Preparation kit (Roche Diagnostics, Mannheim, Germany)	18S rRNA	Nested PCR	159	Pf/Po (3) Pf/Pm (1) Total = 4	Pf/Pm/Po (2)
4.	Camargo-Ayala et al. [5]	Colombia (2012–2015)	Febrile patients (671)	< 5, 5–18, 18–60, and > 60 years	Triple infection: < 5 (4), 5–18 (2), 18–60 (6), > 60 (2) Dual infection: < 5 (16), 5–18 (56), 18–60 (139), > 60 (15)	EDTA blood	Pure Link Genomic DNA mini kit (Invitrogen)	18S rRNA	Nested PCR	531	Pv/Pm (190) Pf/Pv (25) Pf/Pm (10) Total = 225	Pf/Pm/Pv (14)
5.	Dhangadamajhi et al. [6]	India (2008)	Febrile and asymptomatic (242)	0–5 years, 6–15, and > 15 years	Triple infection: 0–5 (1), 6–15 (2), > 15 (2) Dual infection: 0–5 (7), 6–15 (32), > 15 (39)	EDTA blood	Phenol–Chloroform extraction	18S rRNA	Nested PCR	197	Pf/Pv (15) Pf/Pm (54) Pv/Pm (10) Total = 79	Pf/Pm/Pv (5)
6.	Dormond et al. [40]	Switzerland (2004–2008)	Malaria positive (89)	>16 years		EDTA blood	MagNA Pure LC DNA isolation kit (Roche, Basel, Switzerland)	18S rRNA	Nested PCR	89	Pf/Po (3) Pf/Pm (2) Total = 5	Pf/Pm/Po (1)
7.	Fuehrer et al. [41]	Bangladesh: Chittagong Hill Tracts (2007–2008) Malaria Research Initiative Bandarban field site (2008–2009)	Febrile patients (379)	Any age		Dried blood spots	Modified Chelex-based technique, the InstaGene Whole Blood Kit (Bio-Rad Laboratories, Hercules, CA)	18S rRNA	Nested PCR	189	Pf/Pv (21) Pf/Pm (2) Total = 23	Pf/Pm/Pv (2)
8.	Gabrielli et al. [42]	Congo (2014)	Residents (306)	1 week to < 5 years		Dried blood spots	The commercial kit Dried Blood spot DNA isolation Kit (Norgen Biotek Corp, Ontario, Canada)	18S rRNA	Nested PCR	164	Pf/Pm (13) Pf/Po (2) Pf/Pv (1) Total = 16	Pf/Pm/Pv (1)
9.	Hopkins et al. [22]	Uganda (2010–2011)	Residents (272)	5–81 years: 5–10, 11–20, and ≥ 21 years		EDTA blood	Phenol–Chloroform extraction	18S rRNA	Nested PCR and qPCR	199	Pf/Pm (38) Pf/Po (8) Total = 46	Pf/Pm/Po (10)
10.	Jiang et al. [43]	Myanmar (2008)	Malaria positive (146)	NA		Dried blood spots	Chelex extraction kits	18S rRNA	Nested PCR	146	Pf/Pk (13) Pv/Pk (13) Pf/Pv (10) Total = 36	Pf/Pv/Pk (2)
11.	Kasehagen et al. [11]	Papua New Guinea (2001–2003)	Residents (16,209)	< 2, 2.0–3.9, 4.0–6.9, 7.0–9.9, 10.0–19.9, 20.0–39.9, and ≥ 40 years		EDTA blood	QIAamp 96 DNA Blood Kit (QIAGEN, Valencia, CA)	18S rRNA	LDR-FMA	658	Pf/Pv (72) Pf/Pm (32) Pf/Po (13) Pv/Pm (17) Pv/Po (9) Pm/Po (4) Total = 147	Pf/Pm/Pv (28) Pf/Pv/Po (5) Pf/Pm/Po (4) Pv/Pm/Po (4) Pf/Pv/Pm/Po (11)
12.	Krishna et al. [18]	India (2015)	Doubtful microscopic (355)	≤ 1, > 1–4, >4–8, > 8–14, and > 14 years		Dried blood spots	FavorPrep Genomic DNA Mini Kit (Favorgen Biotech Corp., Taiwan).	18S rRNA	Nested PCR	353	Pf/Pv (59) Pf/Pm (3) Total = 62	Pf/Pm/Pv (5) Pf/Pv/Po (1) Pf/Pv/Pm/Po (1)
13.	Lorenzetti et al. [44]	Brazil (2003–2005)	Malaria positive (115)	18–52 years		EDTA blood	Phenol–Chloroform extraction	18S rRNA	Nested PCR	115	Pf/Pm (2) Pf/Pv (28) Total = 30	Pf/Pm/Pv (1)
14.	Marques et al. [45]	Mozambique (2001–2002)	Residents (308)	1–82 years		EDTA blood	Phenol–Chloroform extraction	18S rRNA	Nested PCR	115	Pf/Pm (70) Pf/Po (10) Total = 80	Pf/Pm/Po (9)
15.	May et al. [48]	Nigeria (1996–1997)	Residents (593)	Children in Abanla (1–11 years), Children in Ibadan (3–8 years), Children in health centres in Ibadan (0.8–11 years), Healthy adults (15–56 years)		EDTA blood	DNA-Easy Kit (Invitrogen, St. Louis, MO)	18S rRNA	Nested PCR	165	Pf/Pm (41) Pf/Po (12) Total = 53	Pf/Pm/Po (27)
16.	Mehlotra et al. [13]	Papua New Guinea (1998–1999)	Residents (1848)	All groups < 1–85 years: 2–4, 5–9, ≥ 10 years	Triple infection: 2–4 (2), 5–9 (8), ≥ 10 (24) Dual infection: 2–4 (14), 5–9 (51), ≥ 10 (115)	EDTA blood	QIAamp 96 spin blood kits (QIAGEN, Valencia, CA)	18S rRNA	Nested PCR	541	Pf/Pv (116) Pf/Pm (27) Pf/Po (11) Pv/Pm (21) Pv/Po (4) Pm/Po (3) Total = 182	Pf/Pm/Pv (23) Pf/Pv/Po (9) Pf/Pm/Po (1) Pf/Pv/Pm/Po (3)
17.	Mehlotra et al. [12]	Papua New Guinea (1996)	Malaria positive (173)	5–10 and > 11 years	Triple infection: 5–10 (1), > 11 (0) Dual infection: 5–10 (6), > 11 (9)	EDTA blood	QIAamp 96 or individual spin blood kits (QIAGEN, Valencia, CA)	18S rRNA	Nested PCR	163	Pf/Pv (40) Pf/Pm (16) Pf/Po (4) Pv/Pm (3) Total = 63	Pf/Pm/Pv (27) Pf/Pv/Po (8) Pf/Pm/Po (5) Pv/Pm/Po (1) Pf/Pv/Pm/Po (9)
18.	Mueller et al. [14]	Papua New Guinea (2005)	Residents (2527)	< 10 and ≥ 20 years		EDTA blood	QIAmp 96 DNA Blood kits (Qiagen, CA)	18S rRNA	A semi-quantitative post-PCR, ligase detection reaction/fluorescent microsphere assay (LDR-FMA)	1844	Pf/Pv (363) Pf/Pm (136) Pf/Po (27) Pv/Pm (26) Pv/Po (7) Pm/Po (1) Total = 560	Pf/Pm/Pv (99) Pf/Pv/Po (33) Pf/Pm/Po (8) Pv/Pm/Po (6) Pf/Pv/Pm/Po (17)
19.	Nino et al. [46]	Colombia (2015–2016)	Febrile patients (1392)	NA		EDTA blood	Pure Link Genomic DNA mini kit (Invitrogen)	18S rRNA	Nested PCR	596	Pf/Pv (111) Pv/Pm (340) Pf/Pm (29) Total = 480	Pf/Pm/Pv (52)
20.	Pati et al. [19]	India	Febrile patients (1589)	Severe malaria (15–65 years)		Dried blood spots	Chelex extraction kits	18S rRNA	Nested PCR	110	Pf/Pv (11) Pf/Pm (4) Pv/Pm (3) Total = 18	Pf/Pm/Pv (5)
21.	Perandin et al. [20]	Italy	Febrile patients (122)	NA		EDTA blood	The High Pure PCR template preparation kit (Roche, Indianapolis, Ind.)	18S rRNA	Nested PCR	62	Pf/Po (1)	Pf/Pm/Po (1)
22.	Peruzzi et al. [21]	Italy (2005–2006)	Febrile patients (139)	2–49 years	Triple infection: 19 years	EDTA blood	High Pure PCR Template Preparation Kit” (Roche)	18S rRNA	Nested PCR	36	None	Pf/Pm/Po (1)
23.	Pongvongsa et al. [28]	Laos-Vietnam border (2010)	Residents (3059) Sample for PCR (135)	*P. knowlesi* (2–15 years)	Triple infection (Pf/Pv/Pk): mean 6.43 years (7) Dual infection (Pk/Pv): mean 7.4 years (5)	Dried blood spots	QIAamp DNA micro kit (QIAGEN, Tokyo, Japan)	18S rRNA	Nested PCR	90	Pf/Pv (15) Pf/Pm (2 Pv/Pk (5) Total = 22	Pf/Pm/Pv (1) Pf/Pv/Pk (7)
24.	Putaporntip et al. [25]	Thailand (2006–2007)	Febrile patients (1874)	1–81 years		Dried blood spots	Qiagen DNA Mini Kit	18S rRNA	Nested PCR	1751	Pf/Pv (200) Pf/Pm (6) Pf/Po (2) Pf/Pk (5) Pv/Pm (8) Pv/Po (4) Pv/Pk (4) Pm/Po (1) Total = 230	Pf/Pm/Pv (4) Pf/Pv/Po (7) Pf/Pv/Pm/Po (1)
25.	Rubio et al. [38]	Spain (1997–1998)	Febrile patients (168)	NA		EDTA blood	Modified Chelex-based technique, the InstaGene Whole Blood Kit (Bio-Rad Laboratories, Hercules, CA)	18S rRNA	Nested PCR	89	Pf/Pm (4) Pf/Po (1) Pf/Pv (3) Total = 8	Pf/Pm/Pv (1)
26.	Rubio et al. [39]	Equatorial Guinea (1996)	Febrile patients (159)	< 6 years		Dried blood spots	5% Chelext-100 Resin (Bio-Rad Laboratories, Hercules, CA)	18S rRNA	Nested PCR	126	Pf/Pm (36) Pf/Po (3) Pf/Pv (2) Total = 41	Pf/Pv/Po (3)
27.	Sitali et al. [29]	Zambia (2012)	Residents (873)	< 6 years		Dried blood spots	Chelex extraction kits	18S rRNA	Nested PCR	474	Pf/Pm (31) Pf/Po (10) Pf/Pv (1) Total = 42	Pf/Pm/Po (6) Pf/Pm/Pv (1)
28.	Sluydts et al. [15]	Cambodia (2012)	Residents (5793)	2–5, 5–14, 15–39, and ≥ 40 years		Dried blood spots	Instagene^®^ Matrix resin (Bio-Rad, Singapore)	18S rRNA	Two-step Real-time PCR	368	Pf/Pv (56) Pf/Pm (5) Pf/Po (1) Pv/Pm (3) Pv/Po (11) Total = 76	Pf/Pm/Pv (4) Pf/Pv/Po (1) Pf/Pv/Pm/Po (1)
29.	Steenkeste et al. [16]	Cambodia (2001)	Residents (337)	NA		Dried blood spots	the Instagene resin (Bio-Rad, USA)	18S rRNA	Nested PCR	140	Pf/Pv (52) Pf/Pm (15) Pf/Po (4) Total = 71	Pf/Pv/Po (7) Pf/Pm/Pv (24) Pf/Pv/Pm/Po (8)
30.	Steenkeste et al. [17]	Cambodia (2001)	Residents (134)	< 1– > 60 years: < 1, 1, 2, 3, 4, 5–9, 10–14, 15–19, 20–39, 40–60, and > 60 years		Dried blood spots	Instagene resin (Bio-Rad, Germany) a	18S rRNA	Nested PCR	102	Pf/Pm (4) Pf/Po (2) Pf/Pv (18) Pv/Pm (3) Total = 27	Pf/Pm/Pv (7) Pf/Pv/Po (3) Pf/Pv/Pm/Po (3)
31.	Subissi et al. [23]	Uganda (2010)	Residents (509)	1–5, 6–10, and > 20 years	Triple infection: 1–5 (5), 6–10 (5), > 20 (0) Dual infection: 1–5 (27), 6–10 (24), > 20 (2)	Dried blood spots	QIAamp mini kit (QIAGEN, Venlo, The Netherlands)	18S rRNA	Nested PCR	299	Pf/Pm (39) Pf/Po (14) Total = 53	Pf//Pm/Po (10)
32.	Toma et al. [27]	Laos (1997)	Residents (336)	< 11– > 50 years: 2–68 years in Phavang and 0–75 years in Sisomsouen		EDTA blood	GFX Genomic Blood DNA Purification Kit (Pharmacia Biotech).	18S rRNA	Nested PCR	117	Pf/Pv (19) Pf/Pm (2) Pv/Pm (2) Pv/Po (1) Total = 24	Pf/Pm/Pv (1) Pf/Pv/Po (1) Pf/Pv/Pm/Po (1)
33.	Woldearegai et al. [47]	Gabon (2016)	Residents (834)	1–96 years: 1–5, 6–10, 11–15, 16–20, 21–25, 26–30, 31–40, 41–50, 51–60, 61–70, 71–80, and 81–96 years		EDTA blood	QIAsymphony DSP DNA kit	18S rRNA	Nested PCR	618	Pf/Pm (123) Pf/Po (43) Pm/Po (1) Total = 167	Pf//Pm/Po (51)
34.	Zhou et al. [24]	Thailand (1995–1996)	Residents (548)	NA		Thick smear	30% Chelex-100	18S rRNA	Nested PCR	114	Pf/Pv (10) Pf/Pm (6) Pv/Pm (19) Pv/Po (1) Total = 36	Pf/Pm/Pv (49) Pf/Pv/Po (2) Pv/Pm/Po (1) Pf/Pv/Pm/Po (7)
35.	Zhou et al. [26]	China (2008–2012)	Febrile patients (562)	NA		Dried blood spots	QIAamp DNA Mini Kit (QIAGEN China (Shanghai)	18S rRNA	Nested PCR	384	Pf/Pv (67) Pv/Po (2) Pf/Pk (2) Pf/Pm (2) Po/Pm (1) Total = 74	Pf/Pv/Po (3) Pf/Pm/Pv (1) Pv/Pm/Po (1) Pf/Pv/Pm/Po (1)
										Total = 12,023	2 species = 3059 Pf/Pv = 1318 Pf/Po = 188 Pf/Pm = 775 Pf/Pk = 26 Pv/Pm = 645 Pv/Po = 39 Pv/Pk = 58 Pm/Po = 10 Pm/Pk = 1	3 species = 601 Pf/Pm/Pv = 355 Pf/Pv/Po = 83 Pf/Pm/Po = 137 Pf/Pv/Pk = 11 Pv/Pm/Po = 13 Pv/Pm/Pk = 2 4 species = 53

Pf refers to *P. falciparum*

Pv refers to *P. vivax*

Po refers to *P. ovale*

Pm refers to *P. malariae*

Pk refers to *P. knowlesi*

*NA* Not Assessed

Regarding the number of malaria-positive participants by PCR, 12,023 patients were infected by one of the five *Plasmodium* species (*Plasmodium falciparum, Plasmodium vivax, Plasmodium malariae, Plasmodium ovale*, and *Plasmodium knowlesi*). Among those positive patients, 3059 (25.4%) were infected with two different *Plasmodium* species. The most common types of mixed infection were *P. falciparum* and *P. vivax* (1318, 11%), *P. falciparum* and *P. malariae* (775, 6.4%), and *P. vivax* and *P. malariae* (645, 5.4%). Among those 12,023 positive patients, 601 (5%) were infected with three different *Plasmodium* species. The most common types of mixed infection were *P. falciparum*/*P. malariae*/*P. vivax* (355, 3%), *P. falciparum*/*P. malariae*/*P. ovale* (137, 1.1%), and *P. falciparum*/*P. vivax*/*P. ovale* (83, 0.7%). Fifty-three patients (0.44%) had quadruple mixed infection with *P. falciparum*/*P. vivax*/*P. malariae/P. ovale*.

Quadruple mixed infection was identified among the present studies and comprised of *P. falciparum*/*P. vivax/P. malariae*/*P. ovale* in one individual. Fifty-three patients (0.44%) had quadruple mixed infection. Most (40/53, 75.5%) were found in Papua New Guinea [11–14], followed by Cambodia (12/53, 22.6%) [15–17], Thailand (8/53, 15%) [24, 25], India (1/53, 1.9%) [18], China (1/53, 1.9%) [26], and Laos (1/53, 1.9%) [27].

### Quality of the included studies

All of the included studies were rated with a maximum of 7 stars (Table 2). Sixteen studies received 7 stars, 12 received 6 stars, and 7 received 5 stars. The twelve studies rated with 6 stars used febrile controls, and the 7 studies rated with 5 stars used malaria-positive samples for PCR analysis.

**Table 2:** Quality of the included studies

No.	References	Selection				Compatibility	Exposure			Total score (7)
		Is the case definition adequate?	Representativeness of the cases	Selection of controls	Definition of controls		Ascertainment of exposure	Same method of ascertainment for cases and controls	Non-response Rate	
1.	Asua et al. [2]	*			NA	*	*	*	*	5
2	Barber et al. [3]	*			NA	*	*	*	*	5
3.	Calderaro et al. [4]	*	*		NA	*	*	*	*	6
4.	Camargo-Ayala et al. [5]	*	*		NA	*	*	*	*	6
5.	Dhangadamajhi et al. [6]	*	*		NA	*	*	*	*	6
6.	Dormond et al. [40]	*			NA	*	*	*	*	5
7.	Fuehrer et al. [41]	*	*		NA	*	*	*	*	6
8.	Gabrielli et al. [42]	*	*	*	NA	*	*	*	*	7
9.	Hopkins et al. [22]	*	□	□	NA	*	*	*	*	7
10.	Jiang et al. [43]	*			NA	*	*	*	*	5
11.	Kasehagen et al. [11]	*	*	*	NA	*	*	*	*	7
12.	Krishna et al. [18]	*			NA	*	*	*	*	5
13.	Lorenzetti et al. [44]	*			NA	*	*	*	*	5
14.	Marques et al. [45]	*	*	*	NA	*	*	*	*	7
15.	May et al. [7]	*	*	*	NA	*	*	*	*	7
16.	Mehlotra et al. [13]	*	*	*	NA	*	*	*	*	7
17.	Mehlotra et al. [12]	*			NA	*	*	*	*	5
18.	Mueller et al. [14]	*	*	*	NA	*	*	*	*	7
19.	Nino et al. [46]	*	*		NA	*	*	*	*	6
20.	Pati et al. [19]	*	*		NA	*	*	*	*	6
21.	Perandin et al. [20]	*	*		NA	*	*	*	*	6
22.	Peruzzi et al. [21]	*	*		NA	*	*	*	*	6
23.	Pongvongsa et al. [28]	*	*	*	NA	*	*	*	*	7
24.	Putaporntip et al. [25]	*	*		NA	*	*	*	*	6
25.	Rubio et al. [38]	*	*		NA	*	*	*	*	6
26.	Rubio et al. [38]	*	*		NA	*	*	*	*	6
27.	Sitali et al. [29]	*	*	*	NA	*	*	*	*	7
28.	Sluydts et al. [15]	*	*	*	NA	*	*	*	*	7
29.	Steenkeste et al. [16]	*	*	*	NA	*	*	*	*	7
30.	Steenkeste et al. [17]	*	*	*	NA	*	*	*	*	7
31.	Subissi et al. [23]	□	*	*	NA	*	*	*	*	7
32.	Toma et al. [27]	□	*	*	NA	*	*	*	*	7
33.	Woldearegai et al. [47]	*	*	*	NA	*	*	*	*	7
34.	Zhou et al. [24]	*	*	*	NA	*	*	*	*	7
35.	Zhou et al. [26]	*	*		NA	*	*	*	*	6

### The pooled prevalence of triple mixed infection

**Fig. 2:**
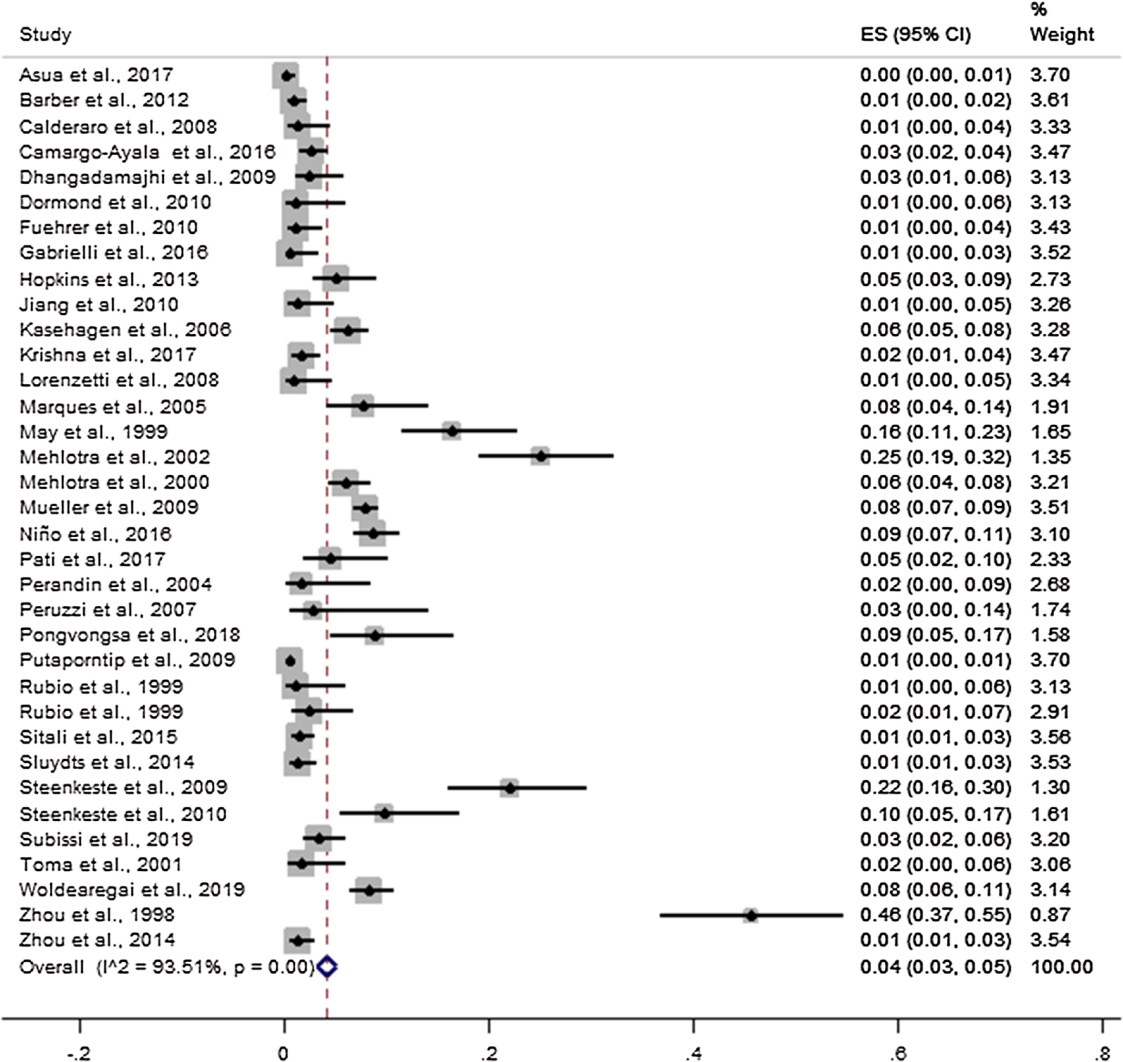
Pooled prevalence of triple mixed infections by *Plasmodium* species

The numbers of triple mixed infection were available for 35 records that included 601 patients from 22 countries. The overall pooled prevalence of triple mixed infection (4%; 95% CI 3–5%; I^2^ = 92.5%) with no evidence of publication bias related to small study effects is shown in the funnel plot (Fig. 2). The highest prevalence of triple mixed infection for an individual study was 46% (95% CI 37–55) in a study by Zhou et al. [24].

### Comparison of the pooled proportion of triple and double mixed infections

**Fig. 3:**
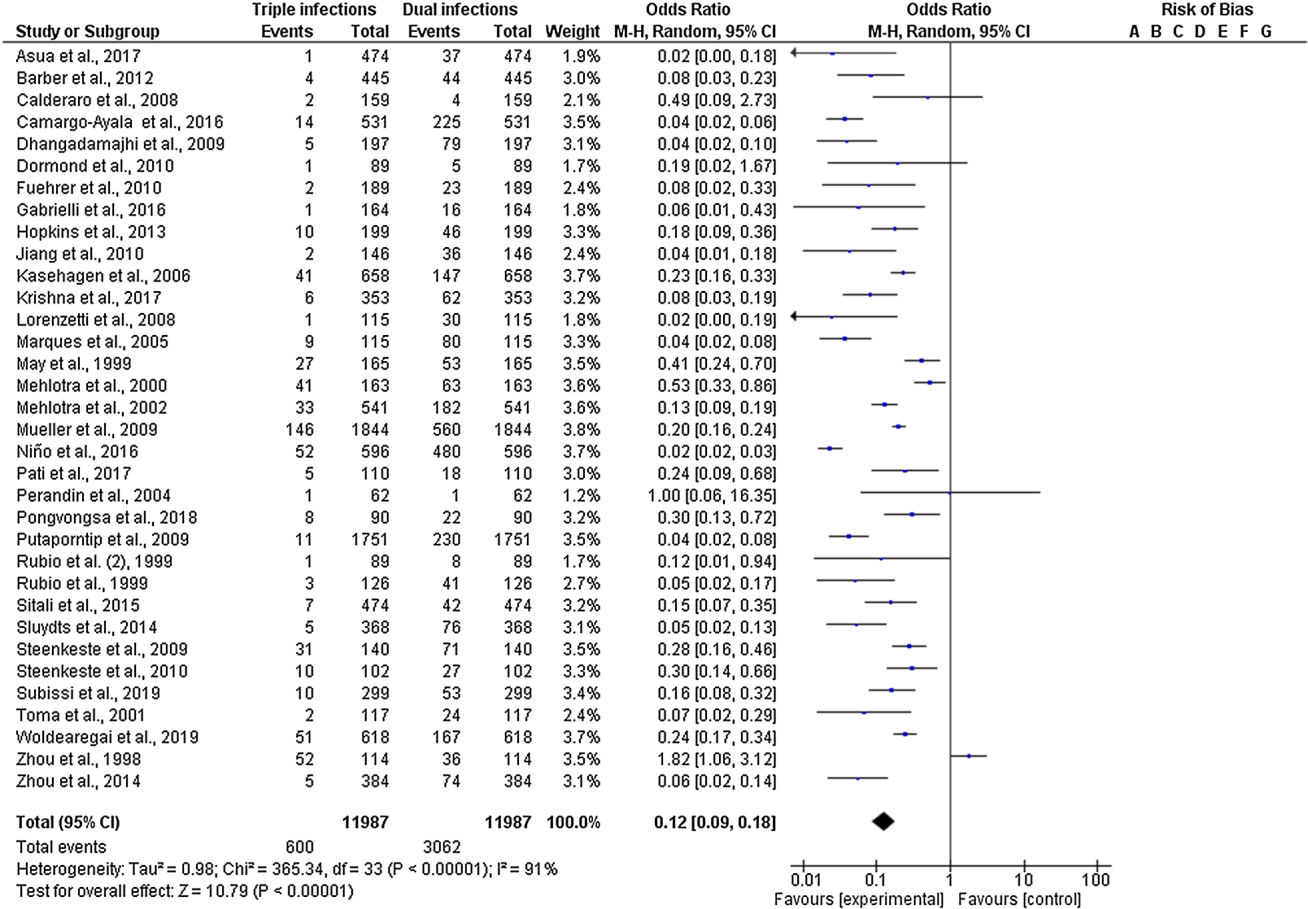
Pooled proportion of triple mixed infections by *Plasmodium* species compared with double mixed infections

The pooled proportion of triple mixed infection compared with double mixed infection was 12% (95% CI 9–18, I^2^ = 91%) (Fig. 3). Most of the included studies (29/35, 82.9%) presented a lower proportion of triple mixed infection than double mixed infection. Only one study demonstrated a higher proportion of triple mixed infection than double mixed infection [24]. Another included study by Peruzzi et al. could not input the present meta-analysis because it had only reported on triple mixed infection, not double mixed infection [21].

### Subgroup analysis

**Fig. 4:**
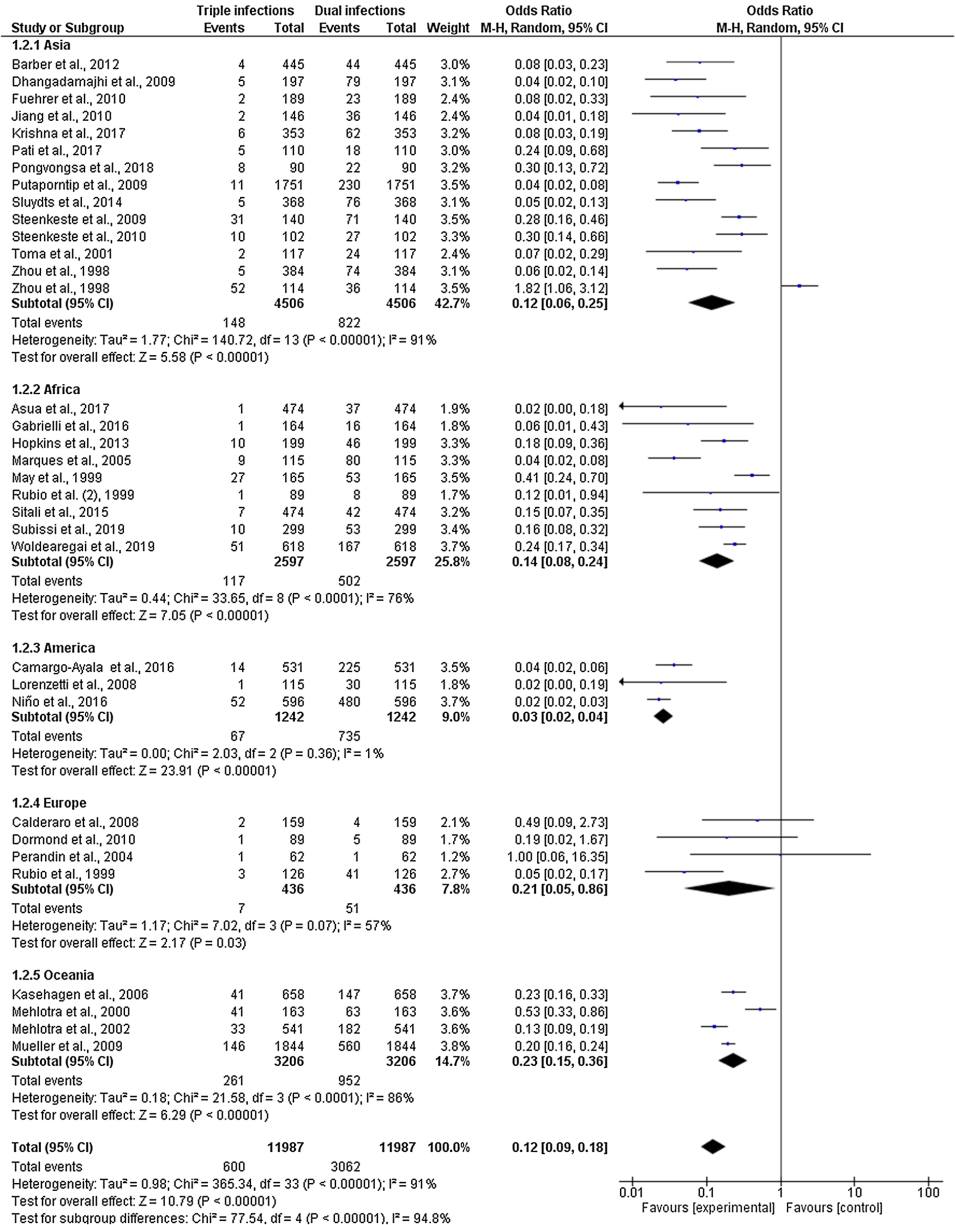
Subgroup analysis of triple mixed infections by *Plasmodium* species between areas of the included studies

Subgroup analysis of the continents from 34 studies where triple mixed infection were reported in the included studies was available. The analysis demonstrated that the proportion of triple mixed infection was the highest in Oceania 23% (95% CI 15–36%) and Europe 21% (95% CI 5–86%) compared with that of double mixed infection (Fig. 4). However, the proportion of triple mixed infection was the lowest in America (3%; 95% CI 2–4%). A subgroup difference was found between continents with a high level of heterogeneity (P-value < 0.0001, I^2^ = 94.8%).

**Fig. 5:**
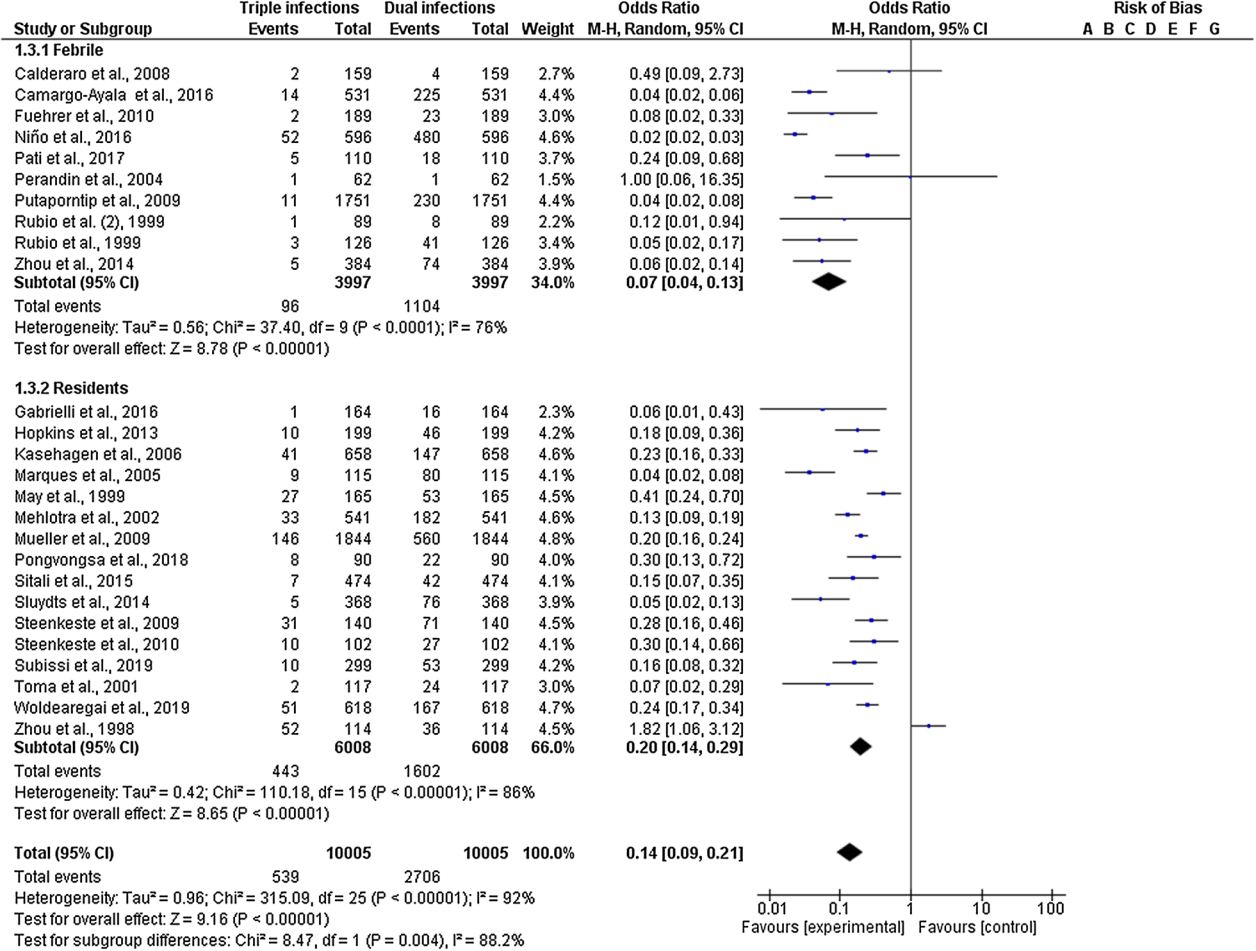
Subgroup analysis of triple mixed infections by *Plasmodium* species between residents and febrile groups

**Fig. 6:**
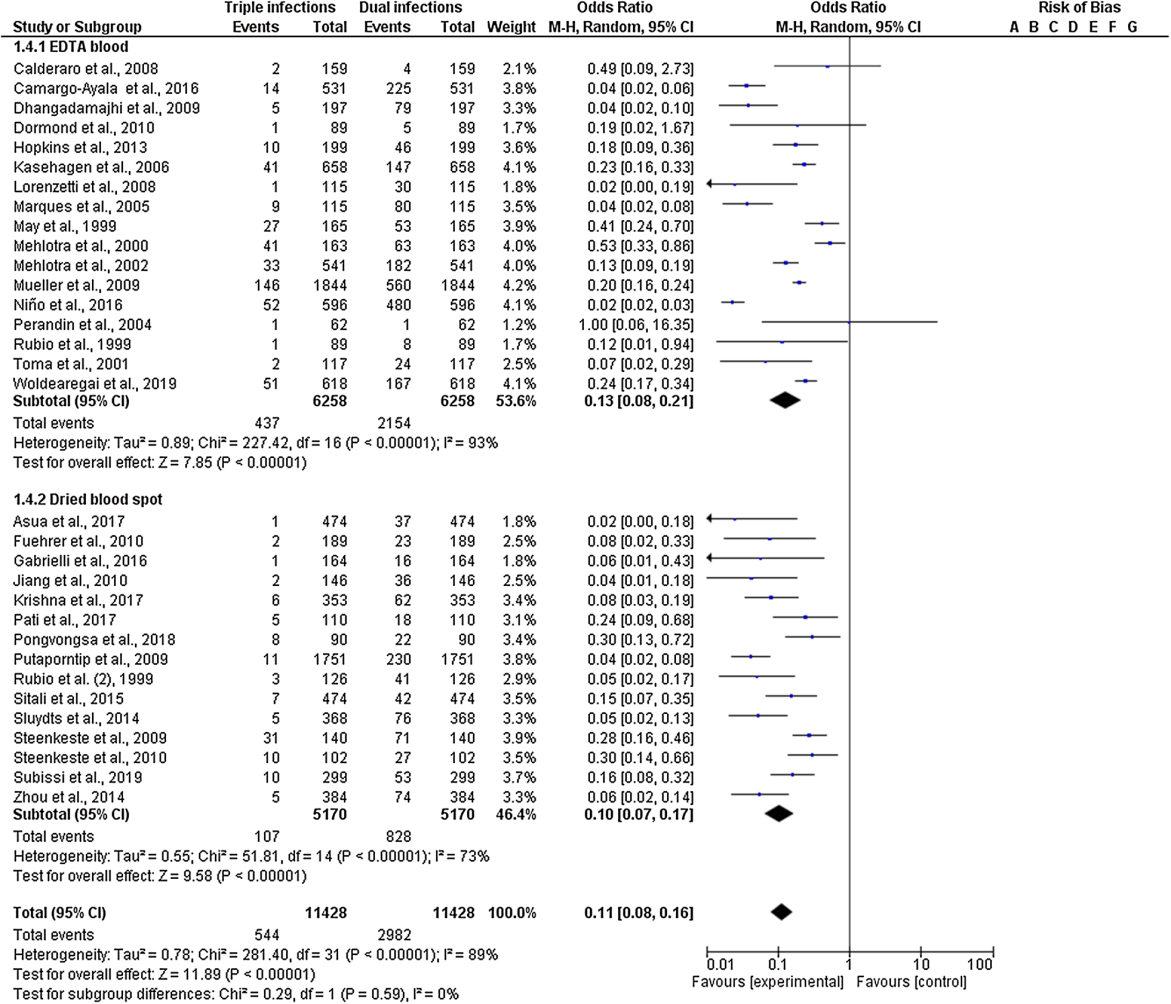
Subgroup analysis of triple mixed infections by *Plasmodium* species between the two blood collection methods

Subgroup analysis of the febrile subjects and residents from 27 studies was available (Fig. 5). Compared with the proportion of double mixed infection, triple mixed infection was higher in residents (20%; 95% CI 14–29%) than in febrile patients (7%; 95% CI 4–13%). A subgroup difference was observed between febrile patients and residents with a high level of heterogeneity (P-value = 0.004, I^2^ = 88.2%). Subgroup analysis of the blood collection method for PCR from 32 studies was available. The proportion of triple mixed infection using EDTA blood was 13% (95% CI 8–21%) and dried blood spots was 10% (95% CI 7–17%), with no subgroup difference between the groups (P-value = 0.59; Fig. 6).

**Fig. 7:**
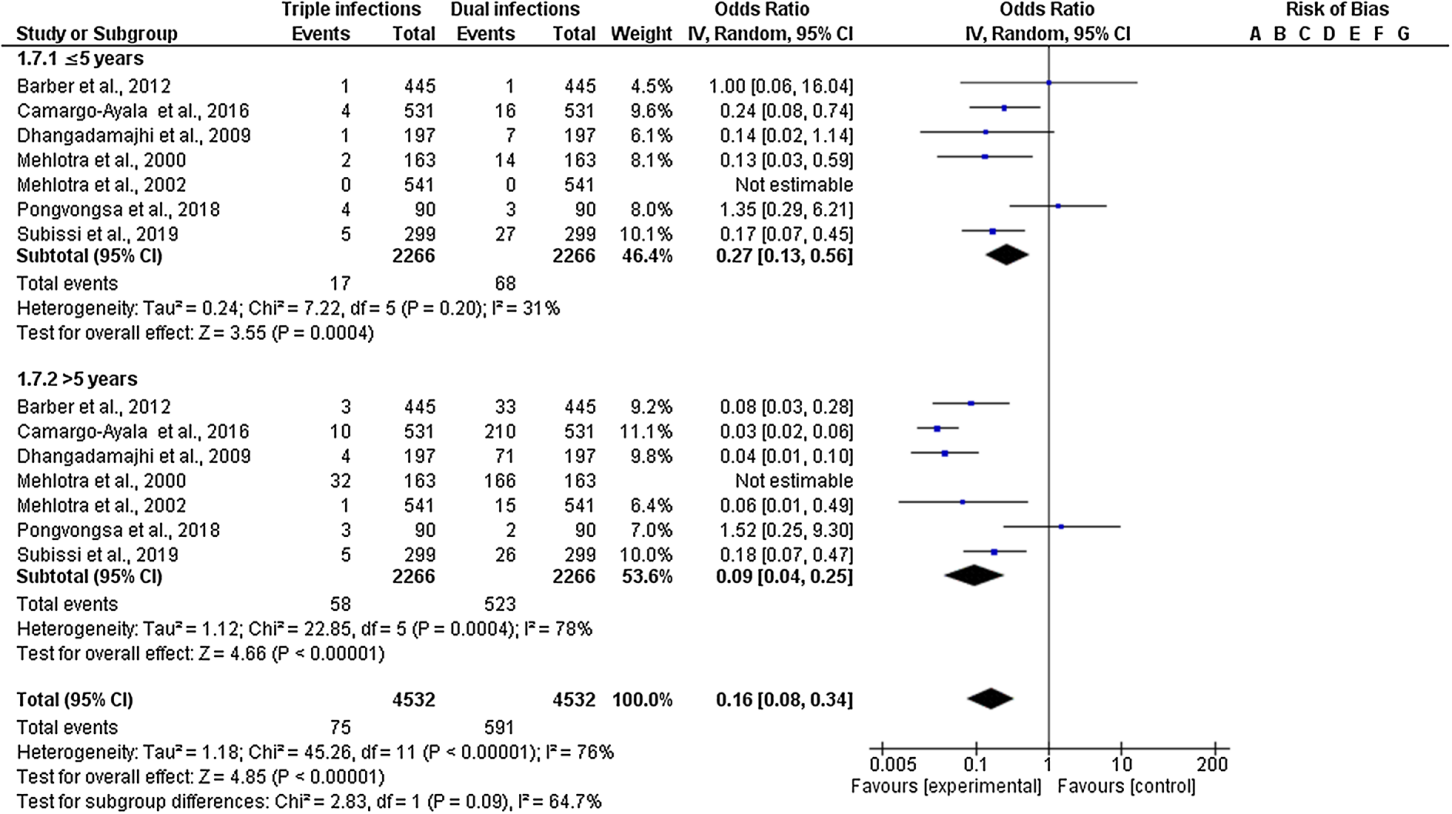
Subgroup analysis of triple mixed infections by *Plasmodium* species between the two age groups

Seven included studies reported the age groups, and different types of mixed infection (85 triple mixed infection and 581 double mixed infection). Subgroup analysis of age groups demonstrated that, compared with the proportion of double mixed infection, triple mixed infection was lower in patients aged ≤ 5 years (OR = 0.27; 95% CI 0.13–0.56; I^2^ = 31%) and > 5 years (OR = 0.09; 95% CI 0.04–0.25; I^2^ = 78%) (Fig. 7). Subgroup analysis demonstrated no statistical difference (P-value = 0.09, I^2^ = 64.7%).

### Publication bias

**Fig. 8:**
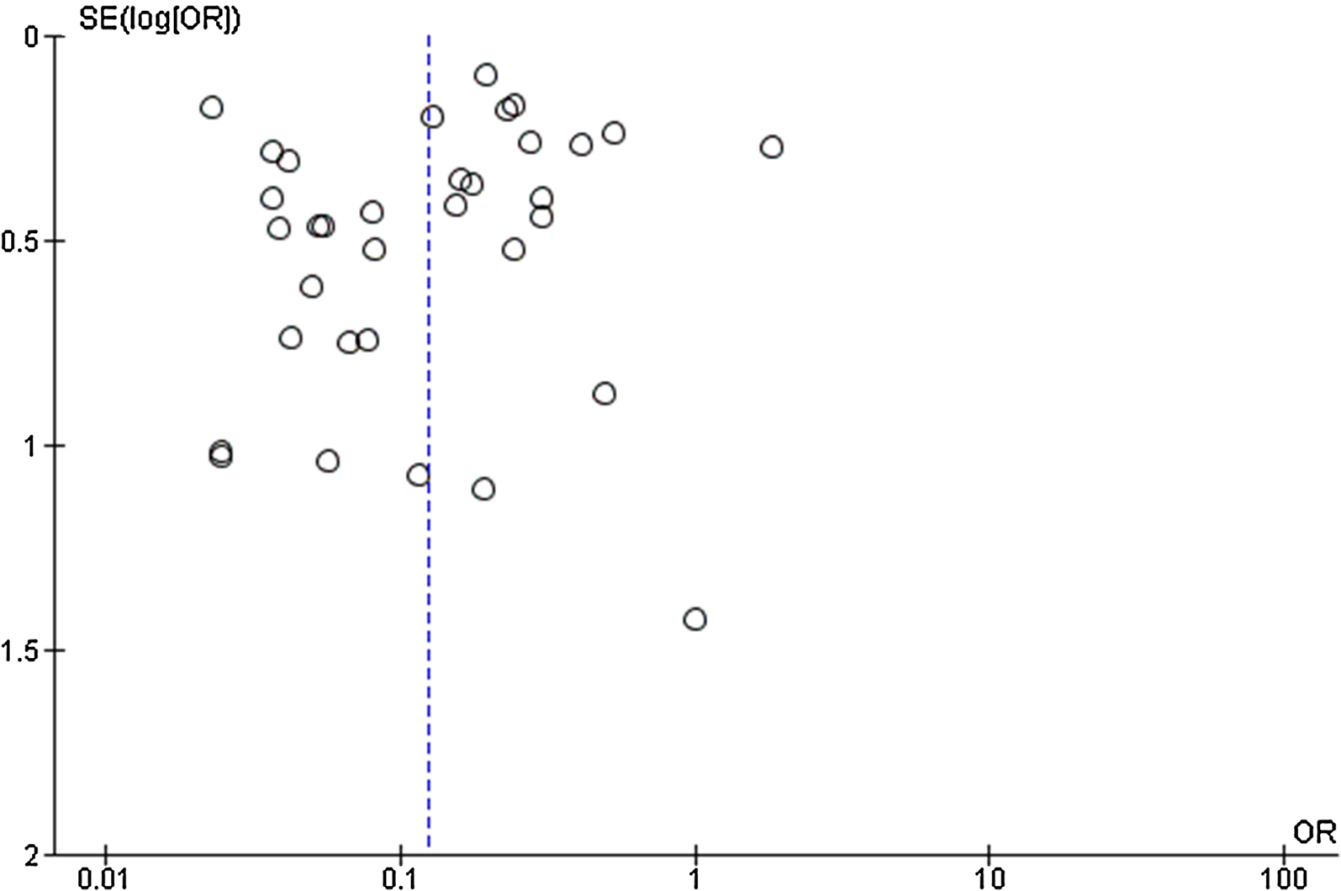
Publication bias among the included studies as demonstrated by funnel plot analysis

Publication bias related to study effects was assessed using funnel plot asymmetry, and no publication bias was demonstrated as evidenced by the symmetry of the funnel plot (Fig. 8).

## Discussion

The pooled prevalence of triple mixed infection has not been clarified in the previous literature. The systematic review and meta-analysis of 12,023 malaria-positive patients demonstrated a high prevalence of triple mixed infection (4%). The most common triple mixed infection of *Plasmodium* species was *P. falciparum*, *P. malariae*, and *P. vivax* (59%). This finding agreed with those of previous studies in Papua New Guinea [11–14], India [18], Cambodia [15–17], and Thailand [24], but contradicts the findings of previous studies which included *P. ovale* in triple mixed infection in the Laos-Vietnam border (*P. falciparum*/*P. vivax*/*P. knowlesi*) [28], China (*P. falciparum*/*P. vivax*/*P. ovale*) [26], Thailand (*P. falciparum*/*P. vivax*/*P. ovale*) [25], and Zambia (*P. falciparum*/*P. malariae*/*P. ovale*) [29].

The mechanisms underlying the triple mixed parasitic infection are unknown; however, the course of an infection might be influenced by the simultaneous occurrence of several *Plasmodium* species [30, 31]. Another explanation is the immunosuppressive effects caused by chronic *P. falciparum* infection and differences in individual exposure [32]. Whether the simultaneous infections might be beneficial or adds further detriment to the infected individual is not well defined. Triple mixed infection may be caused by cross immunity-induced susceptibility to three infections or exposure to infective bites of a single vector that can transmit three *Plasmodium* species [33]. A previous study indicated that infection with one *Plasmodium* species increased susceptibility to infection by other *Plasmodium* species [34]. The apparent frequency of mixed infection is dependent on the technique used for parasite analyses. The results demonstrated a high proportion of triple mixed infection compared with double mixed infection only in studies using PCR analysis to detect the malaria parasite due to the high sensitivity and specificity of PCR compared with microscopy or RDTs. In areas where more than one *Plasmodium* species is present and transmission is stable, the adult populations often have parasite densities below the level of microscopic detection and called “submicroscopic infections”. These submicroscopic infections demonstrated more than one *Plasmodium* species.

The subgroup analysis demonstrated that the proportion of triple mixed infection was higher in residents than in febrile patients, indicating that residents in communities where malaria is endemic were exposed to malaria several times or to more than one species at a time [35]. These triple mixed infection were submicroscopic infection for which microscopy has insufficient sensitivity for their detection. It is well-documented that malaria patients in endemic areas develop immunity against malaria, resulting in symptom relief [35, 36]. A previous study demonstrated that age, geographical origin, and clinical manifestations were found to be associated with triple mixed infection [5]. The subgroup analysis of age ranges demonstrated that the proportion of triple mixed infection was significantly lower across a wide age range of ages compared to double mixed infection. Subgroup analysis demonstrated that no statistical difference in age groups and types of mixed infection. This result suggested that triple mixed infection can occur in both patients aged ≤ 5 years and > 5 years. However, a limited number of articles have reported on age and susceptibility to triple mixed infection. The included study by Camargo-Ayala et al. showed that patients tend to have a risk of triple mixed infection at an age range of 18–60 years than at ≤ 5 or 5–18 years, whereas patients tend to have a risk of double infection at age range greater than 60 years than at ≤ 5, 5–18 and 18–60 years [5]. However, small sample sizes of the triple and dual mixed infection were calculated for the risk estimate in the same study. Therefore, the association between age and different types of mixed infection (double and triple infection) should be analysed in further observational studies using the research gap in age and type of mixed infection.

The study demonstrated that vomiting and the intense brown colour of urine were associated with triple mixed infection. Regarding the geographical region analysed, triple mixed infection was mostly found at the Loretoyacu River in the Colombian Amazon region [5]. The high prevalence of triple mixed infection at the Loretoyacu River may be due to the occurrence of the mosquito *Anopheles maculatus*, which can serve as a single vector for *P. falciparum*/*P. vivax*/*P. malariae* [5]. Triple species infection of *P. falciparum* and *P. malariae*, followed by *P. ovale* delayed infection, were also observed in two adopted children from the Central African Republic and may be attributed to late therapeutic failure or the relatively insufficient dosage due to increased oral clearance of atovaquone in paediatric patients [37].

The subgroup analysis demonstrated that the proportion of triple mixed infection was the highest in Oceania (23%) and Europe (21%) but the lowest in America. A previous study indicated that, in Oceania, where intense transmission occurs in very small focal forests or forest fringe areas, mixed infection are common but require submicroscopic detection [9]. Malaria disease in Europe has been mostly eradicated, but the increase in the number of imported malaria due to tourism, as well as population migration, resulted in increased mortality, from 3.8 to 20% [38]. These imported cases have increased the number of malaria cases in places where its transmission was low or previously eradicated, such as in Europe [39].

Knowledge about mixed infection is important not only to develop appropriate control measures but also for therapeutic options. For example, if *P. vivax* infection is suppressed by mixed infection with *P. falciparum*, effective control of *P. falciparum* infection in an area will activate *P. vivax* transmission in the community, a condition that is more difficult to control [17]. The present study was limited by the heterogeneity of the included studies and should be interpreted cautiously. Thus, the findings of the present study might not necessarily apply to all co-endemic regions. The present study could not extract the age of patients with triple mixed infection due to the lack of data reported in the included studies. Moreover, the clinical data, laboratory data, and treatment data of individual patients with triple mixed infection were also unavailable to extract. These data should be included and declared in malaria studies for its apparent value in cases of review and meta-analyses. Future meta-analyses should assess the cases reported or case series to provide a greater understanding of the factors associated with triple mixed infection.

## Conclusion

In summary, although mixed infection was recognized, the prevalence of triple mixed infection was high (4%). The proportion of triple mixed infection was the highest in Oceania and Europe but lower in America. Compared with the proportion of double mixed infection, triple mixed infection was higher in residents (20%) than in febrile patients (7%). The findings suggested that in some regions, co-endemic for triple mixed infection, PCR, or molecular diagnosis for all residents in communities where malaria is endemic can provide prevalence data and intervention measures, as well as prevent disease transmissions and enhance malaria elimination efforts.

## Supplementary information

**Additional file 1.** Table S1.

## Data Availability

The datasets used during the current study are available without restriction.

## Abbreviations

CI: Confidence interval
DNA: Deoxyribonucleic acid
NOS: Newcastle–Ottawa Scale
OR: Odds ratio
PCR: Polymerase chain reaction
PRISMA: Preferred reporting items for systematic reviews and meta-analyses
RDT: Rapid diagnostic test
WHO: World Health Organization
