# Supplementary material for: Prevalence and proportion of Plasmodium spp. triple mixed infections compared with double mixed infections: a systematic review and meta-analysis
Source: Malar J. 2020 Jun 24;19:224. doi: 10.1186/s12936-020-03292-8 (PMC7315477; doi:10.1186/s12936-020-03292-8)
Supplement: Supplementary file 1 — Additional file 1. Table S1. [file 12936_2020_3292_MOESM1_ESM.docx]

**Search terms**

(("plasmodium"[MeSH Terms] OR "plasmodium"[All Fields]) OR ("malaria"[MeSH Terms] OR "malaria"[All Fields])) AND ("Mixed infections"[All Fields] OR "Triple infection"[All Fields])
